# Density-dependent dispersal promotes female-biased sex allocation in viscous populations

**DOI:** 10.1098/rsbl.2022.0205

**Published:** 2022-08-03

**Authors:** Chedhawat Chokechaipaisarn, Andy Gardner

**Affiliations:** University of St Andrews, St Andrews KY16 9TH, UK

**Keywords:** constant non-disperser principle, density dependence, kin selection, local mate competition, sex allocation, viscosity

## Abstract

A surprising result emerging from the theory of sex allocation is that the optimal sex ratio is predicted to be completely independent of the rate of dispersal. This striking invariance result has stimulated a huge amount of theoretical and empirical attention in the social evolution literature. However, this sex-allocation invariant has been derived under the assumption that an individual's dispersal behaviour is not modulated by population density. Here, we investigate how density-dependent dispersal shapes patterns of sex allocation in a viscous-population setting. Specifically, we find that if individuals are able to adjust their dispersal behaviour according to local population density, then they are favoured to do so, and this drives the evolution of female-biased sex allocation. This result obtains because, whereas under density-independent dispersal, population viscosity is associated not only with higher relatedness—which promotes female bias—but also with higher kin competition—which inhibits female bias—under density-dependent dispersal, the kin-competition consequences of a female-biased sex ratio are entirely abolished. We derive analytical results for the full range of group sizes and costs of dispersal, under haploid, diploid and haplodiploid modes of inheritance. These results show that population viscosity promotes female-biased sex ratios in the context of density-dependent dispersal.

## 1. Introduction

Sex allocation—the apportionment of reproductive resources between male versus female function—represents a fundamental trade-off and provides among the best quantitative evidence for Darwinian adaptation [1]. One particularly successful avenue of study concerns the consequences of genetic relatedness within mating groups, and especially the potential for ‘local mate competition' (LMC) among related males to drive the evolution of female-biased sex ratios [2]. Hamilton's [2] original model of LMC describes a diploid population subdivided into groups of *n* unrelated mothers whose offspring mate among themselves before mated daughters fully disperse to seek their own reproductive opportunities, and suggests that mothers should invest a proportion (*n −* 1)/2*n* of reproductive resources into sons, such that the classic prediction of equal sex allocation [3,4] obtains in the limit of large groups and female bias is favoured in smaller groups. Relaxing the assumption that mothers are unrelated is expected to further promote female bias [5–7].

The simplest mechanism by which mothers may be related is incomplete dispersal, such that sisters have a tendency to remain together while producing their broods. Investigation of this scenario in an infinite, inelastic, island-model setting has revealed that, in fact, the rate of dispersal has no impact on the optimal sex ratio [7,8]. This owes to the exact cancellation of two opposing effects of incomplete dispersal: increased relatedness, which promotes female bias; and competition for reproductive opportunities among related females, which inhibits female bias [7,8]. This striking invariance result has also been shown to apply to the evolution of altruism, with the condition for natural selection to favour an increase in costly helping being exactly the same in a viscous-population setting as in a fully dispersing population [9], and has sparked much theoretical and empirical investigation into the interplay of relatedness and kin competition in social evolution [10].

One mechanism that can disentangle relatedness and kin competition is density-dependent dispersal [11]. If individuals may adjust their dispersal according to local population density, then they are favoured to do so, and indeed their probability of not dispersing is expected to be inversely proportional to the number of individuals in their neighbourhood prior to dispersal, such that the absolute number of non-dispersers is invariant across neighbourhoods [12]. This ‘constant non-disperser principle' [12] means that post-dispersal resource competition is equally intense in all neighbourhoods irrespective of their pre-dispersal densities, such that there is no inhibitory effect of kin competition [11]. Accordingly, population viscosity—by increasing relatedness—promotes altruism [11]. This effect should also apply to other forms of social behaviour, but the consequences of density-dependent dispersal for sex-ratio evolution remain obscure.

Here, we explore the impact of density-dependent dispersal upon the evolution of sex allocation in a viscous-population setting. Specifically, we develop and analyse a kin-selection model to investigate how sex-ratio evolution is modulated by the rate of individually costly density-independent versus density-dependent dispersal, which we also consider to be an evolutionarily labile trait, yielding analytical results and empirically testable predictions for scenarios characterized by haploidy, diploidy or haplodiploidy. We find that, when individuals may condition their dispersal on local density, population viscosity strongly promotes the evolution of female-biased sex ratios.

## 2. Results

### (a) Mathematical model

We assume an infinite population subdivided into patches, with each patch being founded by *n* mated females, and each of these foundresses producing the same, large number of offspring with a sex ratio of her choosing. After rearing their offspring to maturity, all foundresses die, and offspring mate at random within their patches with females mating once and males mating potentially numerous times. After mating, all males die, and each mated female either remains in her natal patch or else attempts to disperse to a randomly chosen patch, with a proportion *c* of dispersers dying *en route*. Following dispersal, *n* mated females are chosen at random within each patch to become the next generation of foundresses, with all other females dying.

### (b) Density-independent dispersal

Assuming that all females disperse at a constant rate *d*, and restricting attention to haploidy and diploidy, we employ a kin-selection analysis [13,14] to show that the optimal sex-allocation strategy (i.e. proportional investment into males) is

**Figure 1.**
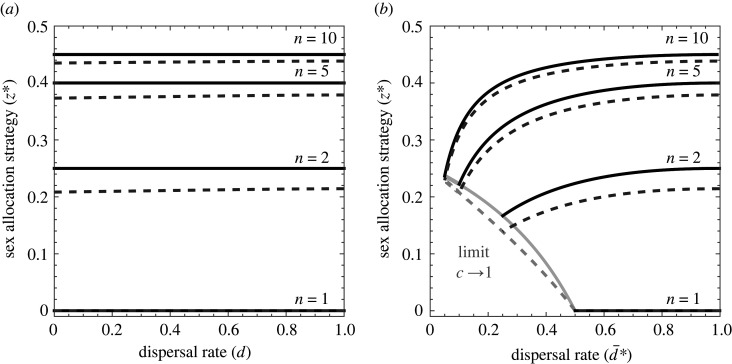
(*a*) Optimal sex allocation is independent of density-independent dispersal rate under haploidy and diploidy (solid lines) and weakly dependent on density-independent dispersal rate under haplodiploidy (dashed lines, with cost of dispersal *c* = 0.5). (*b*) Optimal sex allocation is strongly dependent upon density-dependent dispersal rate, with more-female-biased sex ratios being favoured in more-viscous populations, under haploidy (solid lines), diploidy (solid lines) and haplodiploidy (dashed lines)—and where the cost of dispersal is varied to induce variation in the optimal dispersal rate. Shown are results for patch sizes *n* = 1, 2, 5 and 10.

2.1 $$z^{*}=\frac{n-1}{2n}$$

and hence completely independent of dispersal rate (see electronic supplementary material for details). Although reducing the rate of dispersal leads to higher relatedness, which promotes female bias, it also intensifies competition for reproductive opportunities among related females, which inhibits female bias, and these two effects exactly cancel, such that population viscosity has no net impact upon sex allocation under haploidy or diploidy (figure 1*a*, solid lines). This result was first shown for diploidy—numerically by Bulmer [8] and analytically by Frank [7], for costless dispersal, and analytically by Taylor [15], for costly dispersal—and later for haploidy—analytically by Gardner *et al*. [16], for costless dispersal. Here, it is shown for haploidy and costly dispersal for the first time.

For haplodiploidy, we find that the optimal sex-allocation strategy is

2.2 $$z^{*}=\;\frac{n-1}{2n}\;\frac{4n\;-\;(n-1){\left(\left(1-d)/(1-dc\right)\right)}^{2}-\;2}{4n\;-\;(n-1){\left(\left(1-d)/(1-dc\right)\right)}^{2}-\;1},$$

(see electronic supplementary material for details). That is, in contrast to the exact invariance obtained for haploidy and diploidy, the sex-allocation optimum does depend on the rate of dispersal under haplodiploidy, albeit weakly (figure 1*a*, dashed lines). This owes to an inbredness effect—arising in addition to the relatedness and kin-competition effects—whereby consanguinous mating increases the relatedness value mothers place upon daughters, which promotes female bias, and is stronger in more-viscous populations [17]. The result given in equation (2.2) was first shown by Taylor [15].

Here, we have treated the rate of dispersal (*d*) as an arbitrary parameter, which can be varied independently of foundress number (*n*) and cost of dispersal (*c*). However, the rate of dispersal is itself expected to be honed by natural selection, and its optimal value modulated by these more-basic population parameters. If individuals cannot condition their dispersal according to density, then for haploidy, diploidy and haplodiploidy the optimal dispersal rate is given by $d^{*}=2/(1+2cn+{\left(1+4n(n-1)c^{2}\right)}^{1/2})$ [18–21] (see the electronic supplementary material for details). This is of no consequence for sex allocation under haploidy and diploidy, as equation (2.1) is completely independent of dispersal rate. However, substituting this dispersal optimum into the sex-ratio optimum for haplodiploidy, given by equation (2.2), obtains

2.3 $$z^{*}=\frac{n-1}{2n}\;\frac{8n\;-\;2c^{2}(n-1)(3n-2)(3n-1)-\sqrt{1+4n(n-1)c^{2}}-\;3}{8n\;-\;2c^{2}(n-1){\left(3n-1\right)}^{2}-\;2},$$

which is a weakly decreasing function of the cost of dispersal, in line with the expectation that population viscosity weakly promotes female-biased sex allocation under haplodiploidy.

### (c) Density-dependent dispersal

We now turn our attention to the possibility that individuals may condition their dispersal according to local density, i.e. the number of mated females in the patch prior to dispersal, and we investigate the consequences for sex allocation. Denoting by *D* the relative density of a focal female's patch, i.e. the ratio of the number of mated females on her patch prior to dispersal and the average number of mated females per patch across the entire population, we find that for haploidy, diploidy and haplodiploidy the optimal dispersal rate is

2.4 $$d_{D}^{*}=1-\frac{2c^{2}n-1+\sqrt{1+4n(n-1)c^{2}}}{2cn(1+c)D},$$

(see electronic supplementary material for details). The probability 1–*d*_D_* of not dispersing is inversely proportional to patch density *D* and, accordingly, the number of non-dispersers (1–*d*_D_*)*D* within a patch is completely independent of its density—i.e. Crespi & Taylor's [12] ‘constant non-disperser' result. The optimal dispersal rate for a patch of average density—and hence also the overall dispersal rate across the population—is given by substituting *D* = 1 into equation (2.4), and this obtains ${\bar{d}}^{*}=2/(1+2cn+{\left(1+4n(n-1)c^{2}\right)}^{1/2})$, which is the same as given above for density-independent dispersal [11,18–21] (see the electronic supplementary material for details).

Implementing density-dependent dispersal as described by equation (2.4), we find that the optimal sex-allocation strategy under both haploidy and diploidy is

2.5 $$z^{*}=\frac{2n\;-\;1\;-\;\sqrt{1\;+\;4n\;(n-1)c^{2}}}{4n\;(1-c^{2})},$$

which is markedly different from that given by equation (2.1) for density-independent dispersal and, in particular, corresponds to a more-female-biased sex ratio for all intermediate costs of dispersal (0 < *c* < 1; see the electronic supplementary material for details). By varying the cost of dispersal, we may explore a range of dispersal rates and corresponding sex ratios, and this reveals that population viscosity is strongly associated with female bias (figure 1*b*, solid lines). This result owes to the constant non-disperser principle, whereby the production of an extra daughter simply leads to an extra mated female dispersing away from the patch and hence no intensification of kin competition (cf. [11]). Accordingly, the principal effect of a reduction in dispersal is an increase in relatedness, which acts to promote female bias.

For haplodiploidy, we find that the optimal sex-allocation strategy is

$$z^{*}=\frac{\begin{array}{cc} & n(8n-7-5H)+2(1+H)+c^{2}(n-1)(2(1+H)-\\ & n(9n(2n-3-H)+9H+11)) \end{array}}{4n(c^{2}-1)(1+c^{2}{\left(3n-1\right)}^{2}(n-1)-4n)},$$

where *H* = (1 + 4*n*(*n−*1) *c*^2^)^1/2^. This, too, corresponds to a more-female-biased sex ratio being promoted in more-viscous populations (figure 1*b*, dashed lines). As before, owing to the additional inbredness effect, the sex-allocation optimum is somewhat more female biased under haplodiploidy as compared with haploidy and diploidy.

## 3. Discussion

Competition between related males for access to mating opportunities has long been understood to favour the evolution of female-biased sex ratios [2], and the simplest mechanism for ensuring relatedness between social partners is when individuals do not disperse away from their place of origin over the course of their lives [13,22]. Yet the optimal sex-allocation strategy has been shown to be independent of the rate of dispersal in the simplest viscous-population setting [7,8]. This surprising invariant result owes to a cancellation of two opposing effects of incomplete dispersal: increased relatedness, which promotes female bias; and competition among related females for reproductive resources, which inhibits female bias [7,8]. Here, we have shown that when individuals may condition their dispersal behaviour according to local population density they are favoured to do so, and that this leads to female-biased sex allocation being promoted in viscous populations. We have provided analytical results that demonstrate this effect for haploidy, diploidy and haplodiploidy, across the full range of foundress numbers and costs of dispersal, yielding quantitative and qualitative predictions that are amenable to empirical testing.

The Bulmer–Frank sex-allocation invariant [7,8]—and the equivalent result given for altruism by Taylor [9]—is based upon the assumption that all mated females have the same probability of dispersing. Yet, if individuals may facultatively adjust their dispersal to local population density, then they are favoured to do so, and indeed the optimal probability of not dispersing is inversely proportional to density such that the absolute number of non-dispersers is expected to be the same across all neighbourhoods irrespective of this density variation [12]. Kanwal & Gardner [11] have recently shown that this ‘constant non-disperser' phenomenon leads to the kin-competition consequences of altruism (which increases local pre-dispersal density) being completely abolished (on account of the equalizing of post-dispersal densities across the population), such that population viscosity does, in fact, promote the evolution of altruism. In relation to sex allocation, such density-dependent dispersal ensures that the production of extra daughters prior to dispersal is not associated with an intensification of local competition among mated females for reproductive resources following dispersal, as all mated females above the constant threshold disperse to pursue reproductive opportunities elsewhere in the population. Accordingly, we have revealed that population viscosity does, in fact, promote female-biased sex allocation.

The Bulmer–Frank sex-allocation invariant was shown for haploidy by Gardner *et al*. [16], for diploidy by Bulmer [8] and Frank [7], and for haplodiploidy by Taylor [15]. Under haploidy and diploidy the invariance is strict, owing to the exact cancellation of the relatedness and kin-competition consequences of density-independent dispersal (figure 1*a*, solid lines). However, under haplodiploidy the invariant is only approximate, with the overall level of female bias being somewhat stronger under this mode of inheritance and slightly more so in viscous populations (figure 1*a*, dashed lines). This difference owes to an additional inbredness effect [17] that increases the relatedness value mothers place upon daughters under haplodiploidy, and hence leads to further female bias. We observe this same discrepancy in relation to the level of female bias that is favoured under density-dependent dispersal, such that although the sex-allocation invariant breaks down for all three modes of inheritance, the degree of female bias observed under haploidy and diploidy (figure 1*b*, solid lines) is less extreme than under haplodiploidy (figure 1*b*, dashed lines).

We have made several simplifying assumptions in order to facilitate our analysis, and a useful avenue for future theoretical investigation will be to explore the consequences of relaxing these assumptions. We have assumed that all mothers produce the same number of offspring, so it would be useful to explore the impact of fecundity heterogeneity, which is known to lead to breakdown of the Bulmer–Frank invariant [23]. Similarly, we have assumed that each female mates with only one male, and the consequences of female promiscuity—which has been shown to lead to the breakdown of Taylor's [9] altruism invariant [24,25]—remain to be investigated. In addition, we have assumed complete maternal control of sex allocation, yet there may be consequences of fathers having full or partial control of sex allocation (cf. [2,26–29]). We have also assumed that mating occurs before dispersal, and note that sex-allocation optima are expected to be different when mating occurs after dispersal [24,30]. Finally, factors other than density might be expected to modulate a female's dispersal decision-making—such as the dispersal status of her mother, which is a predictor of relatedness to her patch mates [31]. Females might also be able to directly recognize kin, and adjust not only dispersal but also sex-allocation behaviour according to this information. Although a greater inclination to dispersal and female-biased sex allocation is expected in the presence of kin, the impact of kin discrimination on the population-average levels of these social behaviours are more difficult to anticipate [11,32].

Density-dependent dispersal has been observed across diverse taxa, from microbes to mammals [12,33–36]. Empirical studies suggest that sex allocation can be shaped by population density and dispersal status [37–39], yet the possible role for density-dependent dispersal to modulate the evolution of sex allocation remains to be investigated empirically. Species with readily measurable dispersal and sex phenotypes, such as thrips [12,40], may provide opportunities for empirical testing. In addition to comparative study of natural populations, experimental-evolution studies whereby dispersal regimes are imposed on laboratory populations to investigate their impact on sex-ratio evolution [29,41,42] represent an exciting avenue for future research.

## Data Availability

The data are provided in the electronic supplementary material [43].
